# Atopy manifestations in pediatric patients with acute lymphoblastic leukemia: correlation assessment with interleukin-4 (IL-4) and IgE level

**DOI:** 10.1186/s12887-022-03216-2

**Published:** 2022-03-21

**Authors:** Omid Reza Zekavat, Elham Nikpendar, Sezaneh Haghpanah, Negin Shokrgozar, Seyed Javad Dehghani, Nargess Arandi

**Affiliations:** 1 Hematology Research Center, Shiraz University of Medical Sciences, Shiraz, Iran; 2 Neshat Laboratory Research Center, Shiraz University of Medical Sciences, Shiraz, Iran

**Keywords:** Acute lymphoblastic leukemia, Atopy, IL-4, IgE

## Abstract

**Background:**

Acute lymphoblastic leukemia (ALL) is the most common type of cancer in the age range of under 15 years old and accounts for 25–30% of all childhood cancers. Although conventional chemotherapy regimens are used to improve the overall survival rate, it has been associated with some complications, amongst which allergic manifestations with unknown mechanisms are more common.

**Methods:**

Our study compared serum IgE and IL-4 concentration, as a hallmark of allergic responses in pediatric ALL patients before and after 6 months of intensive (high-dose) chemotherapy, to show whether changes in the level of these markers may be associated with atopy. Serum level of IL-4 and IgE was measured using enzyme-linked immunosorbent assay (ELISA) method.

**Results:**

The results showed that the level of IgE and IL-4 increased following chemotherapy in both ALL patients with and without atopy. In addition, post-chemotherapy treatment IgE and IL-4 levels were significantly elevated in patients with atopy compared to those without it. The difference between baseline and post-chemotherapy level of IgE and IL-4 was significantly higher in patients with atopy compared to those without it.

**Conclusions:**

To the best of our knowledge, this is the first study that showed a connection between post-chemotherapy allergic manifestations in pediatric ALL patients and IL-4 and IgE level. Flow cytometry analysis of the T-helper 2 (Th2) lymphocytes and other allergy-related T cell subsets like Tc2 and Th9 as well as the study of the genetic variations in atopy-related genes like IL-4/IL-4R, IL-5, IL-9, IL-13, and high affinity FcεRI IgE receptor and also HLA genes is necessary to clearly define the underlying mechanism responsible for post-chemotherapy hypersensitivity reaction in pediatric ALL patients.

## Background

Acute lymphoblastic leukemia (ALL) is the most common childhood cancer (accounting for about 25–30% of cancers in children under 15 years old) and also the most common type of leukemia (about 80%), characterized by malignant transformation of the lymphoid precursors in the bone marrow [1]. Usually, chemotherapy is used as the standard first line treatment for pediatric ALL. The established treatment protocol includes induction, consolidation, and long-term maintenance, along with CNS prophylaxis given at specified intervals during therapy [1, 2]. Although chemotherapy has greatly improved the clinical outcome of patients, the main barrier is post-chemotherapy adverse events, which potentially affect the efficacy of treatment [1, 2]. Hypersensitivity is the major infusion reaction observed after chemotherapy, which occurs as a result of the immune system activation against chemotherapeutic agents [3, 4]. However, the rate of these reactions has been reduced remarkably by administration of the less immunogenic form of chemotherapy drugs [3, 5, 6]. Nevertheless, some patients still develop hypersensitivity reactions with unknown reason. The underlying mechanism has not been clearly defined, but production of the allergy-promoting mediators by the immune system might be implicated in this phenomenon.

IL-4 is the most common cytokine produced by T-helper 2 (Th2) lymphocytes and the key cytokine that regulates Th2 cell polarization [7, 8]. Signaling delivered through IL-4/IL-4R promotes STAT3 activation followed by activation of c-Maf and GATA-3 Th2-polarizing transcription factors, which further stimulate Th2 cell differentiation and IL-5, IL-13 as well as IL-4 production. Therefore, they potentiate Th2 responses [7, 8]. In addition, IL-4/IL-4R signaling promotes B cell proliferation and stimulates immunoglobulin class-switching to IgE antibody, the major antibody in allergic reactions [7, 8]. Production of these cytokines by Th2 lymphocytes and other cells accounts for the activation of the mast cells, basophiles, eosinophiles and smooth muscle cell contraction as well as stimulation of B cell differentiation into IgE-producing plasma cells, thus promoting several allergic reactions including allergic rhinitis, anaphylaxis, atopic dermatitis, and asthma [7–9]. Until now, there has not been enough data on whether the hypersensitivity events in ALL patients are dependent on the IL-4 and IgE production or not. Therefore, in this study, we aimed to evaluate the allergic manifestations in pediatric patients during intensive (high-dose) chemotherapy and its association with change in the serum IgE and IL-4 levels during this period.

## Methods

### Patients’ characteristics and study design

This is a cohort study in which 39 newly diagnosed untreated pediatric ALL patients who were admitted from May 2019 to January 2021 in Amir Oncology Hospital affiliated to Shiraz University of Medical Sciences were enrolled. All participants had confirmed diagnosis of ALL (B-ALL/T-ALL) by bone marrow aspiration, biopsy, and flowcytometry and had received standard risk or high-risk chemotherapy protocol, which was adjusted by the age and total white blood cell count at presentation. All patients experienced chemotherapy drugs including vincristine, doxorubicin, peg-asparaginase, methotrexate, cytosar, mercaptopurine, thioguanine, and cyclophosphamide during the first 6 months of intensive therapy. Inclusion criteria were newly diagnosed untreated ALL patients with negative history of atopy among them or their first-degree relatives. Exclusion criteria were history of previous treatment with chemotherapy agents for any reasons and/or previous history of any rheumatologic or any chronic diseases, which need regular medical treatment as well as congenital or acquired cellular or humoral immunodeficiency disorders.

Patients were followed through the first 6 months of intensive (high-dose) chemotherapy for any allergic manifestations including allergic rhinitis (AR), upper airway hypersensitivity reaction, asthma, urticaria, and eczema. Accordingly, among the included patients, those who showed allergic symptoms at the end of 6 months high-dose chemotherapy were considered as the atopy ( +) group. The remaining patients who did not present allergic symptoms were known as the atopy (-) group. The laboratory data including white blood cell (WBC) and platelet (Plt) count, percentage of the neutrophils, lymphocytes, and eosinophils as well as serum hemoglobin (Hb) level were measured in all patients at diagnosis and after 6 months of therapy.

### Sample collection

Five milliliters of the peripheral blood were collected prior to chemotherapy onset and before maintenance therapy (about 6 months after intensive chemotherapy treatment). The serum specimens were isolated from the samples by centrifugation (Sigma-Aldrich, USA) of blood samples at 3000 rpm for 5 min; then, they were kept at -80 °C refrigerator until use.

### Quantification of serum IgE

Serum IgE was measured using enzyme-linked immunosorbent assay (ELISA) method (AccuBind®, Monobind Inc., Lake Forest, USA). The sensitivity of the kit was 0.1424 U/ml. The concentration of IgE antibody in unknown samples was calculated based on the standard curve. OD value at 450 nm was measured for all samples by spectrophotometer (BioTek Epoch, UK).

### Quantification of serum IL-4 cytokine

Serum IL-4 was measured by enzyme-linked immunosorbent assay (ELISA) method (Invitrogen, USA), according to the manufacturer’s instruction. The sensitivity of the kit was < 2 pg/ml with assay range (7.8–500 pg/mL) and the specificity was 3% (Intra-assay) and 4.5% (Inter-assay). The OD value at 450 nm was measured for all samples by spectrophotometer (BioTek Epoch, UK). The concentration of IL-4 cytokine in the serum of patients was calculated according to the standard curve.

### Statistical analysis

All data were analyzed using IBM Statistical Package for the Social Sciences (SPSS) version 23. Descriptive data were presented as mean ± standard deviation (SD) and percentages. Comparison of qualitative and quantitative variables was performed by Chi-square test and Student t-test between the two groups of patients, respectively. Comparison of the serum level of IgE and IL-4 at baseline and 6 months after treatment was done by Paired t-test in each group. Pearson correlation coefficient was calculated for the relationship of quantitative variables. *P*-value less than 0.05 was considered statistically significant.

## Results

This study included 39 newly diagnosed pediatric ALL patients; 23 patients (59%) developed post-chemotherapy atopic manifestations. The mean age of the patients was 8.7 ± 3.49 (range 4–15 years) and 9.19 ± 3.97 (range 2–15 years) in the atopy (+) and atopy (-) groups, respectively. The male/female ratio was 14/9 and 9/7 in the atopy (+) and atopy (-) groups, respectively. Presentation of the atopy( +) group included: 6 (26.1%) allergic rhinitis, 7 (30.4%) urticaria, and 10 (43.5%) eczema. The demographic, laboratory, and clinical characteristics of the patients are displayed in Table 1.

**Table 1:** Comparison of demographic, clinical and laboratory characteristics of patients with (atopy +) and without atopy (atopy -)

Groups	Atopy (+) (*n* = 23)	Atopy(-) (*n* = 16)	*P*-value
Parameters			
Age (year)	8.7 ± 3.49	9.19 ± 3.97	0.685
Sex (m/f)	14/9	9/7	> 0.999
**Laboratory data**			
WBC count (× 10^3^)	4.14 ± 1.9	6.87 ± 4.34	0.109
Neutrophil (%)	9.38 ± 9.14	18.42 ± 16.53	0.083
Lymphocyte (%)	82.09 ± 12.27	69.42 ± 18.69	0.38
Eosinophil (%)	1.21 ± 0.53	1.45 ± 0.68	0.288
Plt (× 10^3^)	151.23 ± 36.25	150.64 ± 71.05	0.974
Hb (g/dL)	8.59 ± 1.61	9.13 ± 1.74	0.354
Baseline IgE (IU/ml)	25.85 ± 20.51	21.05 ± 12.85	0.375
Baseline IL-4 (pg/ml)	21.86 ± 7.75	20.84 ± 5.86	0.657
**Clinical characteristics**			
**ALL type**			
B-ALL	16	9	
T-ALL	5	5	
Unknown	2	2	
**Cytogenetic**			
t(10, 14)	2	5	
t(12, 21)	5	1	
t(1, 19)	4	1	
t(4, 11)	1	3	
t(8, 14)	5	1	
t(9, 22)	0	1	
trisomy 4, 10, 17	2	2	
Unknown	4	2	

Data are presented as mean ± SD

### Alteration in the IgE and IL-4 level after chemotherapy in the atopy ( +) and atopy (-) patients

**Fig. 1:**
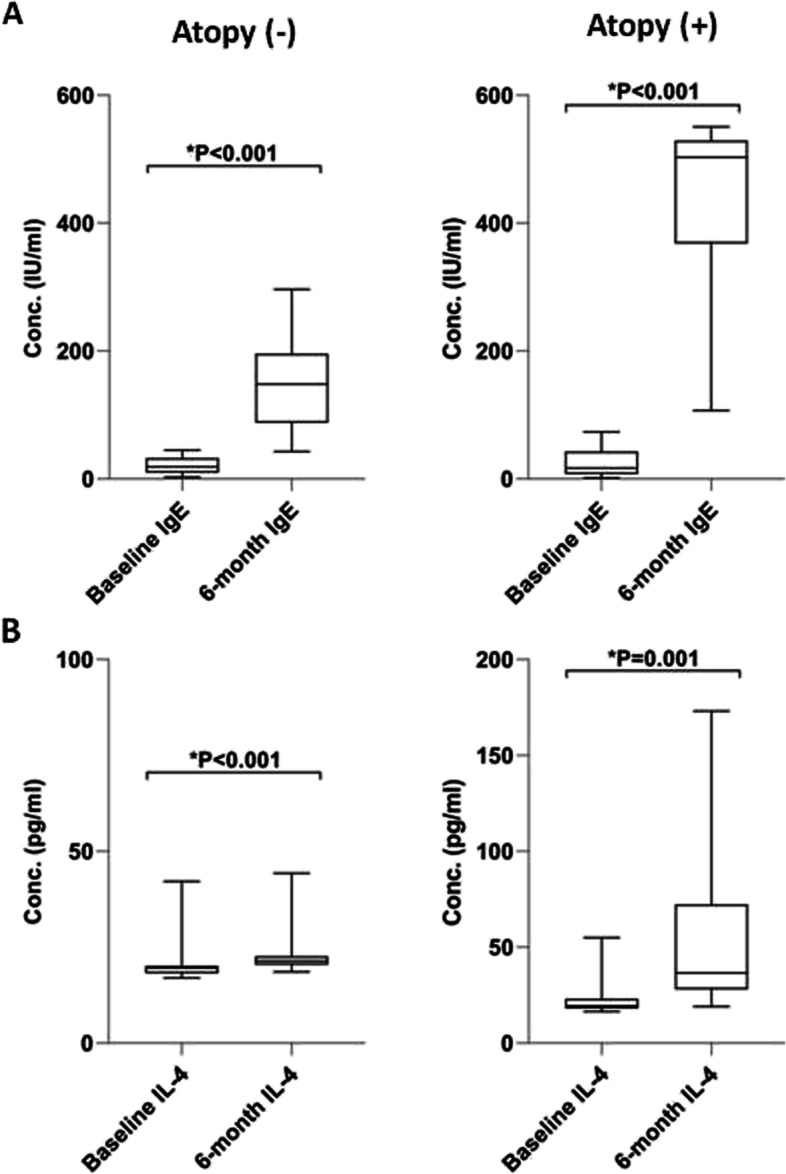
The baseline and 6-month level of IgE (**A**) and IL-4 (**B**) in the atopy ( +) and atopy (-) pediatric ALL patients. The graph is created by GraphPad Prism 8. Data are presented as mean ± SD. *P* < 0.05 is considered as statistically significance. Atopy ( +): patients with atopy, Atopy (-): patients without atopy

The baseline and 6-month value of serum IgE was calculated and compared in the atopy ( +) as well as atopy (-) patients. The results showed that the level of serum IgE significantly increased in the atopy ( +) group after 6 months compared to the baseline level (446.67 ± 113.56 *vs.* 25.85 ± 20.51, respectively; *P < 0.001) (Fig. 1). The IgE level was also elevated after 6 months in the atopy (-) group although with the lesser extent (153.94 ± 79.14 *vs.* 21.05 ± 12.85, respectively; *P < 0.001) (Fig. 1).

Comparison of the IgE level 6 months after chemotherapy indicated that the post-chemotherapy level of IgE was significantly higher in the atopy ( +) compared to the atopy (-) patients (446.67 ± 113.56 vs. 153.94 ± 79.14; *P < 0.001).

The concentration of serum IL-4 was compared between the baseline and 6 months after chemotherapy in both groups. Analysis of the results revealed that, similar to the serum IgE, the level of IL-4 significantly increased post-chemotherapy in comparison to its baseline level in the atopy ( +) group (51.2 ± 35.22 *vs.* 21.86 ± 7.75, respectively; *P = 0.001) (Fig. 1). Moreover, the concentration of the serum IL-4 of the atopy (-) group was significantly raised 6 months after treatment (23 ± 6.04 *vs*. 20.84 ± 5.86, respectively; *P < 0.001) (Fig. 1). Consistent with the serum IgE, the post-chemotherapy level of IL-4 was significantly higher in the atopy ( +) patients compared to the atopy (-) ones (51.2 ± 35.22 *vs.* 23 ± 6.04; *P = 0.001).

### Comparison of the change in the serum IgE and IL-4 between the atopy ( +) and atopy (-) groups

**Fig. 2:**
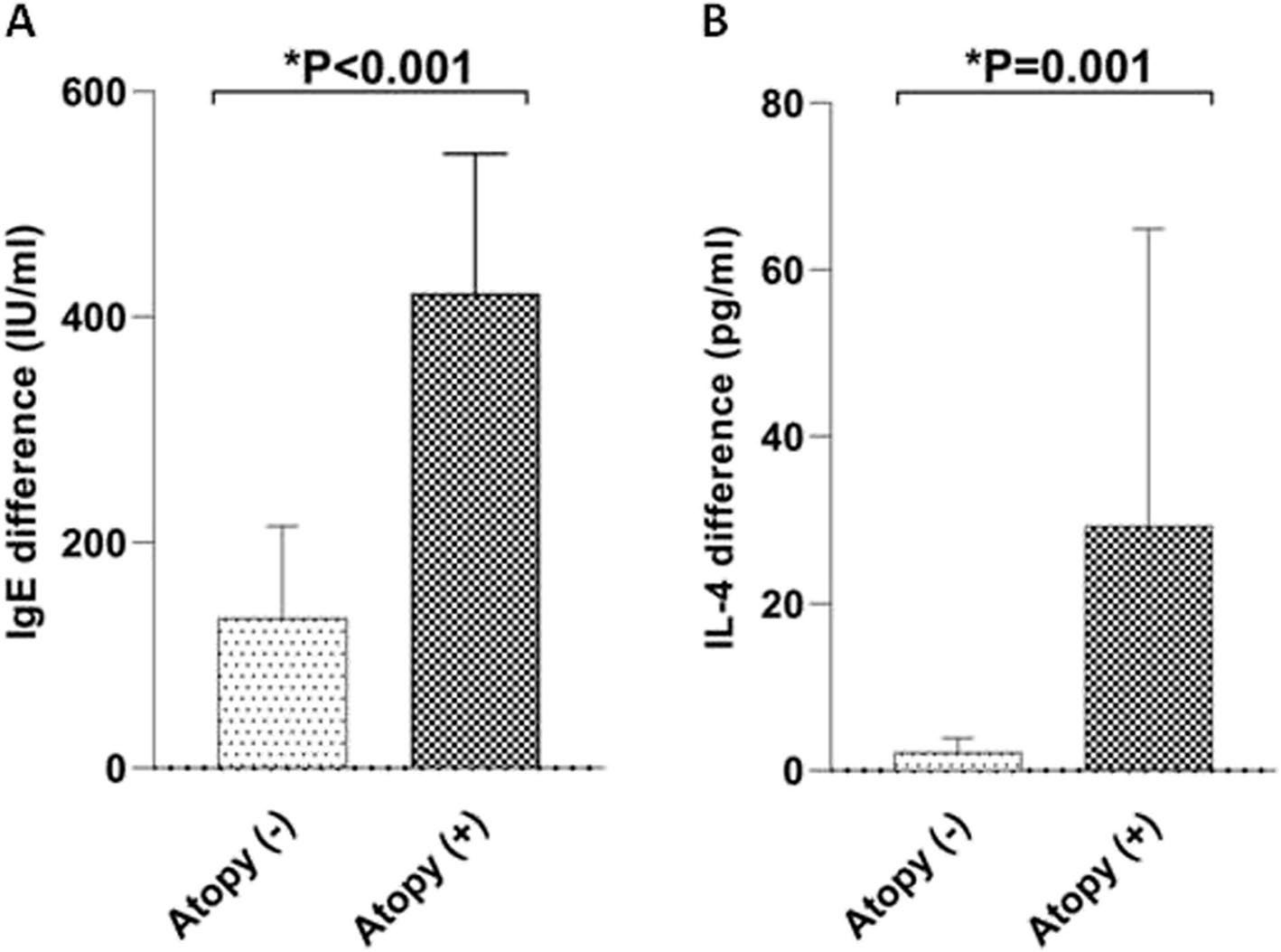
Comparison of the changes in the serum IgE (**A**) and IL-4 (**B**) level between atopy ( +) and atopy (-) pediatric ALL patients. The graph is created by GraphPad Prism 8. Data are presents as mean ± SD. *P* < 0.05 is considered as statistically significant. Atopy ( +): patients with atopy, Atopy (-): patients without atopy

The change in the concentration of serum IgE and IL-4 was calculated by subtraction of their initial value from their post-chemotherapy value and considered as “difference” or “change” in the expression of serum IgE and IL-4 level during this time. Then, the difference in the concentration of the serum IgE and IL-4 was compared between the atopy ( +) and atopy (-) patients. The results demonstrated that the serum IgE level significantly changed in the atopy ( +) compared to the atopy (-) patients following chemotherapy (420.82 ± 124.03 *vs.* 132.89 ± 81.62, respectively; *P < 0.001) (Fig. 2). Similarly, the difference of serum IL-4 was significantly higher in the atopy ( +) patients compared to the atopy (-) group (29.33 ± 5.58 *vs.* 2.16 ± 1.71, respectively; *P = 0.001) (Fig. 2). No correlation was observed between the changes in the serum IgE and IL-4 levels among the atopy ( +) as well as atopy (-) patients (*P* > 0.05).

### Change in the serum IgE and IL-4 level and demographic and laboratory data

The difference in the level of IgE and IL-4 was compared between males and females. Accordingly, the serum IgE and IL-4 alteration did not significantly differ between males and females in patients with and without atopy (*P* > 0.05). No positive correlation was observed between the difference in IgE and IL-4 level and age of patients with and without atopy (*P* > 0.05). In addition, the IgE and IL-4 change did not correlate with laboratory data including WBC and platelet count, percentage of the neutrophils, lymphocytes and eosinophils as well as Hb level in patients with and without atopy (*P* > 0.05).

## Discussion

For many decades, conventional chemotherapy regimen, which is used to improve the overall survival rate in children with ALL, has been connected to different adverse events, amongst which allergic manifestations have gotten more attention [3, 10]. Even though the effector mechanisms are not clearly identified, IgE antibody (antibody-dependent allergic reactions) and other allergy-related mediators including IL-4 cytokine might be involved in the pathogenesis of chemotherapy-related allergic manifestations. In this study, serum IgE and IL-4 levels were evaluated at baseline and after 6 months of chemotherapy as a hallmark of post-chemotherapy allergic susceptibility mediators to show whether changes in their level is associated with hypersensitivity presentations in pediatric ALL patients during high-dose intensive chemotherapy.

Our results showed that the amount of IgE and IL-4 increased after 6 months in both ALL patients with and without atopy compared to the baseline level in each related group, but 6-month post-chemotherapy level of both IgE and IL-4 was significantly higher in the atopy ( +) compared to the atopy (-) group. In addition, changes in the IgE and IL-4 levels after 6 months were significantly higher in the atopy ( +) compared to the atopy (-) group.

Post-chemotherapy hypersensitivity reactions are the commonly observed feature of cancer patients. It is not clear whether changes in the IL-4 and IgE levels in our study are secondary to immune dysregulation in these patients or they are a general reaction against chemotherapy drugs. Although both atopy (+)  and atopy (-) ALL patients actually received the same main treatment protocol, the reasons of why atopy is limited to some patients are unknown.

Obviously, genetic factors like special variants of the IL-4, IL-4R, and IL-13 genes may have a prominent role in development of allergy [11–14]; therefore, their contribution should be carefully noticed. Consistent with this, studies showed that cytokine variants including TNF-α 308 A → G, IL-13 and IL-4RA as well as genetic variation in IgE receptor were associated with predisposition to drug-induced allergy [15, 16]. Interestingly, recent studies revealed that in addition to IgE-mediated drug-induced allergic reactions, differences in major histocompatibility complex (MHC) molecules are the main contributor of T cell-dependent drug-induced allergic manifestations [16]. The types of drugs as well as repeated exposure to chemotherapeutic agents are other factors that have a fundamental role in antibody-mediated allergic reaction and thus, should be taken into account in patients’ management [3, 17].

Therefore, the study of polymorphism in the atopy-related genes including IL-4/IL-4R, IL-5, IL-9, IL-13, IgE receptor, and genetic variations in HLA molecules should not be underestimated and might provide additional data on the exact role of these factors in the development of allergic manifestations in ALL patients. In addition, analysis of IL-4 and IgE concentration at different time points post-chemotherapy especially when patients entered the maintenance phase is required to specify the role of chemotherapy in this phenomenon.

T-helper 2 (Th2) subtypes of CD4^+^ T cells are a subgroup of the lymphocytes which contribute mainly to allergic reactions and immune responses against parasites and helminthes by production of the cytokines IL-4, IL-5, IL-9 and IL-13 that promote B cell proliferation and immunoglobulin class-switching to immunoglobulin E (IgE) [7]. Data on Th2 responses and its related cytokines is very limited in ALL patients and their mechanism of action is poorly understood in these patients. In a study by Zhang et al. on the IL-4-producing CD4^+^ (Th2) and CD8^+^ (Tc2) subpopulations, it was demonstrated that the Th1/Th2 and Tc1/Tc2 ratios were significantly decreased in the peripheral blood T cells of ALL patients (*n* = 30) compared to the healthy controls, suggesting the dysregulated differentiation of Th2 and Tc2 in these patients [18]. Also, Horacek et al. reported a higher IL-4 level in the serum samples of newly diagnosed ALL patients compared to the healthy controls [19]. Stachel et al. showed the increased expression of IL-4 mRNA in the bone marrow of 49 pediatric patients with B cell precursor ALL with late relapse proposing that ALL leukemic cells mediate a shift toward Th2 responses and, thus, influence the relapse risk [20]. Consistent with this, Cardoso et al. revealed that IL-4 positively stimulated the proliferation and growth of T-cell ALL cells by activating mTOR signaling which affects the disease outcome [21]. However, Pérez-Figueroa showed a polarized Th1 cytokine profile (IFN-γ and IL-12) in children with ALL (newly diagnosed) while the level of Th2 cytokines (IL-4 and IL-13) were similar compared to the healthy control group [22].

Our study confirmed that both atopy (-) and atopy ( +) ALL patients developed higher IgE and IL-4 (albeit with the higher extent for atopy ( +) patients) after chemotherapy compared to their corresponding baseline level. Although the reason for this finding is not clear, it could be assumed that the increase in the IL-4 and IgE production in both atopy ( +) and atopy (-) patients might be the result of the dysregulated Th2 responses in these ALL patients. In addition to Th2 lymphocytes, CD8^+^ T cells as well as cells of the innate immune system including the mast cells, eosinophils, basophils, NKT cells and, innate lymphoid cells are also responsible for IL-4 production and IgE class-switching [9, 23]. Accordingly, flow cytometry analysis of Th2 lymphocytes at baseline and after 6 months of chemotherapy can be highly informative and may be necessary to clarify whether Th2 lymphocytes are implicated in the elevation of IL-4 and IgE production in ALL patients and consequently, post-chemotherapy allergic manifestations in the atopy ( +) group. In line with this scenario, it should be noticed that comparison of the frequency of other CD4^+^ subsets that are linked to the allergic diseases like Th9 cells between the atopy (-) and atopy ( +) patients could clearly define the underlying mechanisms responsible for allergic symptoms in atopy ( +) patients. Moreover, mast cells are another compartment of the immune system, which are known as a key driver along with IgE in pathophysiology of allergic reaction [24, 25]. Engagement of FcεRI IgE receptor on the surface of mast cells leads in to mast cell activation and degranulation and thereby, release of inflammatory mediators like histamine, prostaglandins, leukotrienes, cytokines/chemokines, and neutral proteases (including chymase and tryptase) which promote allergic responses [24, 25]. It is tempting that the difference in the mast cell characteristics might be responsible for allergic manifestation in some ALL patients post chemotherapy. This assumption needs to be verified by more studies.

The small number of ALL patients is another limitation of our study that should be taken into account. Accordingly, multi-center studies with higher number of ALL patients could be helpful for better understanding the biological role of IL-4 and IgE as well as other allergy-related mediators in the pathogenesis of post-chemotherapy atopy in ALL patients.

## Conclusion

It is the first time that the higher concentration of IL-4 and IgE has been shown to be associated with post-chemotherapy allergic manifestations in ALL patients. Larger number of ALL patients along with the specific analysis of the Th2 lymphocytes and also other allergy-related subsets like mast cells, Tc2 and Th9 cells is necessary to clarify the role of these cells in post-chemotherapy hypersensitivity reactions in pediatric ALL patients. In addition, study of the genetic variation in IL-4/IL-4R, IL-5, IL-9, IL-13 cytokines, high affinity FcεRI IgE receptor as well as HLA genes and also the evaluation of the level of the cytokines at different time points post-chemotherapy could assist in delineating specifically the underlying mechanism responsible for atopic manifestation post-chemotherapy in some ALL patients.

## Data Availability

The datasets used and/or analyzed during the current study are available from the corresponding author on reasonable request.

## Abbreviations

ALL: Acute lymphoblastic leukemia
ELISA: Enzyme-linked immunosorbent assay
Th2: T helper 2
WBC: White blood cell
Plt: Platelet
SPSS: Statistical Package for the Social Sciences
SD: Standard Deviation
